# The epidemiology of work-related musculoskeletal injuries among chiropractors in the eThekwini municipality

**DOI:** 10.1186/s12998-019-0238-y

**Published:** 2019-03-19

**Authors:** Almay Lamprecht, Keseri Padayachy

**Affiliations:** Department of Chiropractic, Faculty of Health Sciences, Durban University of Technology, Ritson Campus, P O Box 1334, Durban, 4000 South Africa

**Keywords:** Chiropractic, Work-related musculoskeletal injuries, Manipulation, Upper extremity, Low back, Soft tissue injury

## Abstract

**Background:**

Chiropractors are a unique group of health care professionals who are at risk for developing work-related musculoskeletal injuries. Diversity of daily practice imposes different physical demands on the chiropractor. This study aimed to determine the prevalence of work-related musculoskeletal injuries in chiropractors in eThekwini municipality and selected risk factors associated with these work-related musculoskeletal injuries.

**Methods:**

The design was a quantitative, cross-sectional, descriptive study utilising a self-administered questionnaire, developed specifically for this research. The questionnaire contained sections on personal and practice demographics, with questions pertaining to the single most severe work-related musculoskeletal injury, as well as the second and third most severe work-related musculoskeletal injury.

**Results:**

A response rate of 64% was obtained (*n* = 62). The life-time prevalence of work-related musculoskeletal injuries was 69% with a predominance of injuries to the upper extremity (50%) and lower back (28.3%). The hand/wrist was the most common anatomical site of injury (31.5%) followed by the lower back (28.3%). Number of years in practice was considered a risk factor as most injuries occurred within the first five years of practice (41.6%). The majority of injuries affected the soft tissue, including ligament sprains (27.5%) and muscle strains (26.6%) and occurred while the practitioner was performing manipulation (38.2%) of the lumbosacral (80.8%) area with the patient in the side posture (61.5%).

**Conclusions:**

The results concur with other studies on work-related musculoskeletal injuries in chiropractors and add insight into risk factors predisposing this population to injury.

**Electronic supplementary material:**

The online version of this article (10.1186/s12998-019-0238-y) contains supplementary material, which is available to authorized users.

## Background

Work-related musculoskeletal injuries (WRMSI) are a group of painful complaints involving the muscles, tendons and nerves which occurs in an occupational setting due to physical tasks carried out in normal work activities [1–3].

Musculoskeletal disorders may be characterised as episodic disease when pain intensity decreases and increases later on or transient when pain fades with rest or activity modification. However, this depends on the tissue involved and the forces acting upon the body, some musculoskeletal disorders may become persistent or irreversible [1]. Injuries / disorders can be subdivided into occupational loading from long lasting activities occurring over many years during the occupational lifetime or short term loadings resulting predominantly in acute health disturbances whereas long lasting exposure may lead to chronic disorders [4].

Musculoskeletal disorders are further classified as specific or non-specific disorders. Specific musculoskeletal disorders have clear clinical features whereas non-specific musculoskeletal disorders present with pain without evidence of a clear specific disorder.

The Factors that may contribute to musculoskeletal disorders can be grouped into four categories:

- Physical or biomechanical work related factors
- Organisational or psychosocial work related factors
- Individual or personal factors
- Factors relating to social content [1].

Physical factors include work procedures, equipment and environment that lead to biomechanical stress in the muscle, tendons, inter-vertebral discs and nerves. Principle physical work related risk factors in relation to musculoskeletal disorders encompasses force, repetition, awkward position/ posture or long term static postures, vibration and working in low temperatures.

Kumar [5] estimated that approximately 50% of the world’s working population performs hazardous occupations. Performing such occupations requires substantial physical exertion, considerable amount of repetition of those activities and substantial amount of repetition of those activities together with significant time spent in static postures. These unnatural behaviours place the mind and body under tremendous physical and psychosocial stress.

The main cause of disorders/ injuries affecting muscles, tendons, joints, ligaments and bones are attributed to mechanical overload of the respective biological structures [4, 6] . Probable overload of tissues results from high intensity forces or torque acting on and inside the body. The muscles and tendons of the arm are loaded when manual force is used. Repetitive work may cause fatigue and injury when the same muscles and tendons are used for a substantial part of the working day. When placed in awkward postures joints are more susceptible to injuries and muscles have less capacity to exert force. The combination of repetitive, forceful work in an awkward posture poses as a risk factor for the development of work related musculoskeletal injuries [1].

In addition to mechanical overload the duration of exposure primarily determined by the number of repetitions per day as well as total exposure time (hours per day or days per month) are important factors in the development of musculoskeletal disorders [4].

Tissues are overloaded when placed in awkward, constrained, asymmetric, repeated or prolonged postures which exceed the threshold of tolerable stresses of the tissue causing subsequent injury. When muscles contract by-products are created which are removed by the blood [2]. Blood vessels within the muscles are compressed when placed in static postures for prolonged periods of time causing micro-lesions in the muscle due to decreased oxygenation and nutrition and the build-up of by-product [7]. Tendons within sheaths are dependent on the production of lubricating fluid to ensure proper function, with excessive or monotonous movement the lubrication system may falter resulting in friction between the tendon and the sheath leading to the development of tenosynovitis. A ganglion cyst may form if the tendon sheath swells up with lubrication fluid. When tendons are continuously stretched micro-tears can develop leading to tendonitis [1]. Thus, incorrect working posture leads to imbalance and fatigue or over-exertion which cause mostly muscle, tendon and ligament injuries that may result in discomfort and low back pain [7].

Research conducted in the United Kingdom found that musculoskeletal conditions comprise 55% of all work related illness. Acute back pain was the second most highly ranked cause of short term absenteeism among manual workers [9]. The same study found musculoskeletal disorders as the second most commonly identified cause of long term absence for manual workers (44%) closely followed by chronic back pain (42%) [9].

Musculoskeletal disorders account for approximately 33% of all absenteeism from work in industrialised countries. Back related injuries are estimated to be the cause of 60% of absenteeism followed by neck and upper extremity injuries. It is generally accepted that working conditions and work load are important factors for the development and continuance of these disorders [10].

In 2014 a median of 13 days of recuperation were required for workers sustaining a musculoskeletal injury in comparison to 9 days for other types of injuries. While sustaining a fracture, required a median of 32 days to recuperate before returning to work [8]. Most of these musculoskeletal disorders include sprains and strains as result of overexertion in lifting [8]. While a study conducted in Europe found musculoskeletal disorders as a leading cause of temporary and permanent incapacity across Europe. Musculoskeletal disorders accounted for 49.9% of all absenteeism from work lasting more than 3 days or longer and for 60% of permanent work incapacity. The study revealed participants lost an average of 246.6 min of work during the week preceding their participation in that study. An average work week was calculated to 1914 min; the time lost due to musculoskeletal disorders accounted for almost 13% of the work week [9].

The leading types of injuries or illnesses for both males and females were sprains, strains, tears or soreness and pain. Males sustained sprains, strains or tears at a greater rate than females (41.7 cases per 10,000 full time workers compared to 35.8 cases per 10,000 for females). Females incurred bruises and contusions at a greater rate than males with an incidence rate 10.0 compared to a rate of 8.3 for males [8]. It was found that females had a higher incidence rate and number of injuries and illnesses associated with repetitive motion compared to males [8].

The chiropractic profession involves constant performance of various forms of manipulative therapy and other manual tasks in a variety of working postures, which subject the musculoskeletal system to potentially large repetitive mechanical loads.

Manipulation is defined as a manual procedure that incorporates a direct thrust to generate movement in a joint beyond the physiological range of motion short of surpassing the anatomical limit [11]. More precisely, an adjustment is a chiropractic therapeutic procedure that uses precise force, leverage, direction, amplitude and velocity concentrated at specific joints or anatomical regions. Chiropractors influence joint and neurological function by employing these procedures [11]. Adjustments are most commonly applied to the spinal column, but may also be used in the treatment of the extremities and temporo-mandibular joint [12]. Manipulative skills encompasses a collection of psychomotor movement patterns requiring several years of study and training [13, 14]. Achieving good manipulative skills benefits both the patient who receives an effective pain-free manual intervention along with the chiropractor who will evade unnecessary injury and maintain an extensive professional career. To produce focused and localised manipulative thrust suitable body posture and sophisticated bimanual manoeuvres should be learnt [14]. Lauren [15] found that a lack of coordination, strength and effective coupling of the musculature may potentially impair postural stability. Accordingly chiropractors with a smaller physique may possibly have an increased chance of injuring their shoulder and upper back during the performance of more physically demanding manipulative procedures especially with larger patients placed in the side lying position [16].

Chiropractic techniques Cooperstein and Gleberzon [16] estimated that within the chiropractic profession roughly 300 discrete chiropractic techniques are used worldwide. The most commonly applied manipulative procedure is that of the diversified techniques, of which there is roughly 500 separate and distinct manipulations when applying a chiropractic adjustment to a specific anatomical site [14].

The application of spinal manipulative therapy is an active process whereby forces are produced and transferred by means of the upper body and shoulder through the arm and hand [17]. It is important to note that the hand does not contribute to the applied force; the hand acts only as a contact and transfer point. The hand has at least 12 areas which can be used to contact anatomical levers on the patient [14, 18–22] i.e.: pisiform, hypothenar, metacarpal, calcaneal (heel), thenar, thumb, interphalangeal and fingertip(pad).

During a manipulation the hand is the most important short lever contact point used. The hand has the capability to accommodate numerous posturers required to suite the particular clinical situation and patient as well as the capacity to twist and mould to conform to more inaccessible anatomical contact points [23]. Triano [17] found the hand to be susceptible to unnecessary injury if incorrectly placed during the application of the manipulative thrust placing added stress on the soft tissue and joints of the hand and fingers.

Manipulations are performed with the patient in various positions. These positions are determined by symptoms, individual needs,  tolerances and clinical scenarios. Both the side lying and prone posture utilises the shoulder/ arm thrust [22, 24–27]. This specific thrusting technique has the ability to generate large forces over an extended distance. The manipulative force is generated in the shoulder girdle transmitted along the arm across the hand and transferred onto a moderately short anatomical lever [23]. The amount of force applied is considerably influenced by the patient position. The energy used and the force applied is inversely proportioned to the ability to control and stabilise patient movement. The side lying posture exhibits less control and accordingly more force in general. Conversely the prone position offers nearly total patient control, but attaining optimal joint tension is more difficult; possibly increasing the preload forces and compromising specificity [23]. Several authors view the side lying posture as one of the more traditional and most effective positions for the treatment of the lumbar spine and pelvis [14, 22, 28]. The side posture provides leverage via the femur, pelvis and upper body of the patient to produce a mechanical transition point at the desired intervertebral level [17]. This posture subjects both the patient and the practitioner to excessive twisting action which could lead to mechanical deformation of pain sensitive structures.

Chiropractors display an assortment of physical parameters during spinal adjustment [29, 30]. Forces applied to the sacroiliac joint in a side lying position fluctuated between 0 and 300 N preload and 200–1200 N for peak thrust force [23]. A study conducted by Drover [31] compared forces applied by male and female chiropractors during thoracic spine manipulations. The study concluded that from a mechanical point of view female chiropractors delivered similar manual treatments to their male colleagues. The study indicated that a thrust of up to 1000 Newton’s is applied to the target site within approximately 150 milliseconds [31]. An analysis into the three dimensionality of direct contact forces in chiropractic spinal manipulative therapy proposes that the highest loads are at T4–5 and T8–9 levels and the lowest loads at the cervical levels, with T1–2 and sacroiliac loads between both extremes [32].

Aside from manipulation chiropractors regularly use various non-manipulative techniques, commonly referred to as mobilisations. Mobilisations can be defined as a movement applied singularly or repetitively within or at the physiological range of motion, without imparting a thrust impulse, with the objective to re-establish joint mobility [11]. The distinguishing feature between manipulative and non-manipulative techniques is the application of a thrust force. Non-manipulative techniques may not cause as much biomechanical stress to the chiropractor’s hands as a manipulation in a singular event, but the repetitive nature of non-manipulative techniques may have a greater cumulative effect.

Many chiropractors are predisposed to the development of musculoskeletal injuries prior to beginning their professional careers [33]. This may be attributed to performing repetitive adjusting techniques by the novice chiropractic student, leading to upper extremity injuries. Spinal injuries may result from receiving adjustments by inexperienced students [34–36]. All these are predisposing factors for future injury. The continual use of similar manipulative techniques and procedures day after day and year after year could lead to the development of chronic overuse syndrome as the result of poor biomechanical performance by the chiropractor [37].

Daily practice encompasses continuous application of several manipulative procedures and non-manipulative tasks in an assortment of postures which subject the musculoskeletal system to potentially large repetitive mechanical loads [23]. The continual use of similar manipulative techniques and procedures day after day and year after year could lead to the development of chronic overuse syndrome as the end result of poor biomechanical performance by the chiropractor [37]. According to the literature factors related to the administering of manual procedures (e.g. adjustments; massage and motion palpation) have been implicated in the development of unspecified back pain and other occupational injuries in chiropractors [16, 38, 39].

Non-physical stress factors such as financial concerns and patient demands may independently contribute to the commencement of occupational related back pain.

Occupational posture has previously been identified as a predisposing factor for back, neck and shoulder pain [40].

Many manipulative skills utilised in daily practice force the practitioner to assume a bent (flexion) posture, twist (rotation) the trunk, generate a pulling action while simultaneously reaching and stretching around the patient which all predispose the chiropractor to possible WRMSKI [23]. The combination of forward flexion, lateral flexion and rotational movements positions the spinal joints at the end of their passive range, which could result in injury over a period of time as consequence of fatigue or trivial uncontrolled movements [41]. Another risk factor is the constant lifting and readjusting patients on the table prior to the manipulation. Musculoskeletal pain and injuries may be exacerbated by chiropractors modifying their position to meet the patient’s requirements as opposed to adapting the patient’s position in line with their own needs [42].

Soft tissue of the shoulder, elbow and wrist are equally at risk as result of faulty posture and inappropriate force transmission along the kinematic chain resulting in potential occupational related injuries [43]. High patient workload subjects the upper extremity to considerable mechanical loads. The soft tissue of the upper back and shoulder girdle are especially susceptible to injury during manual thrusting as results of the high loads encountered [23]. This could justify and contribute to the high incidence rate of overuse injuries in the chiropractic profession.

Byfield, Maher and McCarthy [43] investigated the prevalence of neck and shoulder pain in the Chiropractic profession in the United Kingdom and found 50% of the sample (*n* = 88) complained of current neck or shoulder pain with 5.7% indicating shoulder and neck pain. Results showed that the cervico-thoracic region was the most common area of neck complaints. Both male (48%) and female (68%) participants felt that their work aggravated their pain.

Homack [44] studied the occupational injuries in practicing chiropractors in the New York State and established that anatomical structures most at risk of being injured were the low back, shoulder and the wrist. The most commonly reported type of injury was muscular strain followed by ligamentous strain. The most common cited cause of injury included patient handling and performing side lying manipulations.

In 2004 Rupert and Ebete [39] conducted a study on the epidemiology of occupational injuries in chiropractic practice with at least 15 years of practice experience. They found that 57% (*n* = 451) of respondents reported work-related musculoskeletal injuries during their career. These musculoskeletal injuries were distributed as follows: wrist (52%), hand (50%), lower back (50%), shoulder (35%), neck (22%) and upper back (21%). The type of injuries reported included ligament strains (45%), muscle strains (43%), tendinitis (37%), vertebral disc (26%) and degeneration (23%). Eighty-two percent of the respondents stated that these injuries caused them to alter activities such as work position (64%), body mechanics (50%), delegated to other personnel (38%) and frequency of manual techniques (33%). In this particular study (*n* = 451) 62% of the participants described modifying patient care due to their symptoms, specifically treatment technique (53%), reduced the number of patients treated (21%) and reduced working hours (18%) as result of injuries encountered.

Holm and Rose [45] determined the prevalence of work-related injuries of chiropractors in the United States (*n* = 159) and found that upper extremity injuries were most commonly reported comprising of wrist/ hand /fingers (42.9%), shoulder (25.8%) and elbow (11.9%). Low back injuries were reported by 24.6% of the respondents. The majority of the injuries included soft tissue injuries such as ligament sprains (44.4%), tendonitis (35.5%) and muscle strains (32.5%). Most of the reported injuries occurred while either positioning a patient for manipulation (11.1%) or while performing a manipulation (66.7%). The areas manipulated whilst sustaining the injury included lumbosacral spine (37.1%) and the thoracic spine (21.6%). These injuries occurred most commonly with the patient being manipulated in the side lying position (37.8%). Furthermore this study showed injuries were more likely to occur in the first to fifth year of practice. With 16.7% of the injuries necessitating at least 1 week or more off from practice and 2.4% resulted in permanent disability. A total of 30% of the participants ($\frac{119}{159}$) indicated a modification to their manipulation technique as result of an injury.

A study conducted by Mathews [46] investigating the prevalence and factors associated with occupational overuse syndrome in the hands and wrists of chiropractors in South Africa (*n* = 108). The study found the lifetime prevalence of either hand or wrist pain in 73% of the participants while 38% had hand and wrist pain. Lumbar spine manipulation caused the most hand or wrist pain in affected participants. The most hand and wrist pain occurred when manipulating patients in the side lying position (46%) followed by having patients lying prone (41%) and supine (35%).

Pereira [47] investigated the prevalence and risk factors for occupational low back pain in manual therapists in South Africa and found that chiropractors (*n* = 21) saw nine patients per day and spent an average of 40 h per week working hands on. Furthermore the study showed that 76.5% of chiropractors (*n* = 17) felt their low back pain was exacerbated by clinical practice. The results showed that 82.4% (*n* = 17) experienced low back pain for the first time working as a manual therapist within 5 years of practice.

There is a higher prevalence of WRMSI in health care workers, which can be attributed to the labour intensive and physically demanding activities required in these professions [48]. Patient handling (including patient transfers, repositioning and lifting) and manual therapy (soft tissue work, mobilisation of joints and orthopaedic techniques) are the activities most commonly cited in association with WRMSI among health care professionals such as physical therapists and occupational therapist [49]. Chiropractors are subjected to lifting, bending and twisting while performing manual therapy; these manual procedures involve rotation as well as forward and lateral flexion of the spine. These movements, combined with awkward positions due to a lack of awareness about their posture [50] cause increased loads on the lower back as well as the upper extremity which are risk factors for the development of work related musculoskeletal injuries [48–51]. The physical demands placed on chiropractors by their occupation places them at risk of developing similar musculoskeletal disorders to those that they treat [52].

The aim of this study was to determine the epidemiology of work-related musculoskeletal injuries among chiropractors in the eThekwini municipality and to compare these findings to similar studies.

## Methods

### Research design

This study was epidemiological in nature; with the aim of establishing patterns in the occurrence of work related musculoskeletal injuries and associating these patterns with likely causes and then quantifying the association [53]. The study was therefore a quantitative, epidemiological, cross-sectional survey, in the form of a self-administered questionnaire. This research was approved by the Durban University of Technology Faculty of Health Sciences Research and Ethics Committee reference number: REC 61/16.

### Participants

A list was obtained from the Allied Health Professionals Council of South Africa (AHPCSA), containing the contact information of all registered chiropractors in the eThekwini municipality. The total population of chiropractors practising in the eThekwini municipality was invited to participate in the study either telephonically or via email whereby they were informed of the particular study, as well as given the opportunity to partake in the study.

The research questionnaire (Additional file 1) was either emailed or hand delivered to the prospective participants together with a Letter of Information and Informed Consent Form. The informed consent requested the chiropractor’s participation and Letter of information explained the purpose of the study as well as the procedure to be followed by participants. The benefits of conducting the research, confidentiality and remuneration were also addressed. Lastly contact details of the researcher and research supervisor were provided should any of the chiropractors have had any queries or questions regarding the study.

The total number of registered chiropractors in the eThekwini municipality was obtained from the AHPCSA on 16 January 2017. It was determined that the registered number of practicing chiropractors in the eThekwini municipality equated to 127 chiropractors.

Target population was 127, only 97 was contactable and of these only 61 agreed to participate. This study was epidemiological in nature; with the aim of establishing patterns in the occurrence of work related musculoskeletal injuries and associating these patterns with likely causes and then quantifying the association [53]. The study was therefore a quantitative, epidemiological, cross sectional survey, in the form of a self-administered questionnaire.

#### Research tool

The questionnaire was adapted from the questionnaire used by Holm and Rose [45]. A study relating to work-related musculoskeletal disorders in chiropractors:

- Questionnaire pertained to Work-related injuries of doctors of Chiropractic in the United States.
  - ο Permission to use the questionnaire was granted by Dr. Kevin Rose

The questionnaire was modified in order to suit a South African audience and in particular the research objectives.

The questionnaire was tested by means of a focus group (FG). The FG consisted of the following members:

- The researcher, who will act as the chairperson of the FG meeting
- The research supervisor who will have guided the researcher through the research process
- Two qualified chiropractors whom have been in practice less than 5 years
- Three qualified chiropractors whom have been in practice more than 5 years

The questionnaire was sent to a statistician prior to the FG meeting as he was unable to attend. The statistician’s comments were raised by the chairperson at the FG meeting. Before starting the FG proceedings, each participant were required to read the Letter of Information (Additional file 1) and sign the Confidentiality Statement, Code of Conduct Statement and Informed Consent Form (Additional file 1). During the course of the FG meeting participants had the opportunity to raise any questions and verify that they comprehend what was required off them. The questionnaire was distributed to each participant and each question in the questionnaire was chronologically discussed by the participants of the FG meeting. Recommended changes were made on the unanimous agreement of the participants. These changes were implemented forming the post- FG questionnaire which was used as the pre-pilot study group questionnaire.

After the FG meeting, the suggested changes were implemented after which the questionnaire was compiled into a post-focus group/ pre-pilot questionnaire. The pilot study served as a “trial run” of the larger study in determining the feasibility of the questionnaire [54–56].

Before starting the Pilot study, each participant was required to read the Letter of Information, sign the Confidentiality Statement, Code of Conduct Statement and Informed Consent Form.

According to Baker [57] enrolling 10–20% of the total sample group is reasonable, thus for this particular study (*n* = 103) 10 participants were enrolled for the pilot study.

The data was collected from the sample of chiropractors in the eThekwini municipality by means of a questionnaire, which was developed from a previously published study [45] and validated prior to the study through a focus group [58, 59] and pilot testing [59].

### Ethics, consent and permissions

Participants participating in the study received a letter of information which introduced the research project by including the title of the study, the aims of the study and re-assuring respondents of the confidentiality of their responses as well as reminding them that their participation was voluntary. Consent was given by each participant.

### Data collection

The research questionnaire was either emailed or hand delivered to the prospective participants. Data collection took place between January 2016 and April 2016. The questionnaire contained sections on personal as well as practice demographics, with questions pertaining to the single most severe work-related musculoskeletal injury, as well as the second and third most severe work-related musculoskeletal injury.

### Data analysis

The data was analysed with SPSS version 24.0. The results present the descriptive statistics in the form of graphs, cross-tabulations and other figures, using the qualitative data collected. The traditional approach to reporting a result requires a statement of statistical significance. A significant result was indicated with “*p* < 0.05”.

Chi-square test was used for nominal and ordinal data at a significance of 0.05, when Chi-square was violated (expected value < 5), Fisher’s Exact Test was used. Binary logistical regression was used to analyse the risk factors of injury.

## Results

Ninety-seven chiropractors were invited to participate in the survey. Seventy-two of them indicated they were willing to participate and 62 chiropractors completed the questionnaire. A response rate of 64% (62/97) was calculated. One unusable response was returned via email. The format of the questionnaire had been altered to a state which could not be utilised for data collection, therefore resulting in the final sample size of *n* = 61.

### Demographics

Of the 61 respondents 27 were male and 34 female. The mean age of respondents was 35.6 years (SD, 8.4 years) (*p* value = 0.078). The mean height was 1.7 m (SD, 0.1) and weight 72.8 kg (SD, 14.7). The number of years in chiropractic practice ranged from less than a year to 40 years, with an average of 9.4 years. The mean practice volume reported was 8 patients per day (SD, 5).

Diversified technique was the most common technique used by 93.4% of chiropractors on a regular basis, followed by Neuro-impulse protocol (NIP) (10.9%), Thompson (5.7%), activator (5.6%) and Gonstead (5.6%) techniques. Majority of respondents used adjunct therapies such as dry needling (75.4%), massage therapy (61.7%), electro-modalities (28.8%) and cryotherapy/ heat therapy (22.8%).

### Injuries

The percentages presented below show the percentage of injuries in the given sample size. Whereas, the *P* values are representative of a comparison between the most, second and third injury options.

**Fig. 1:**
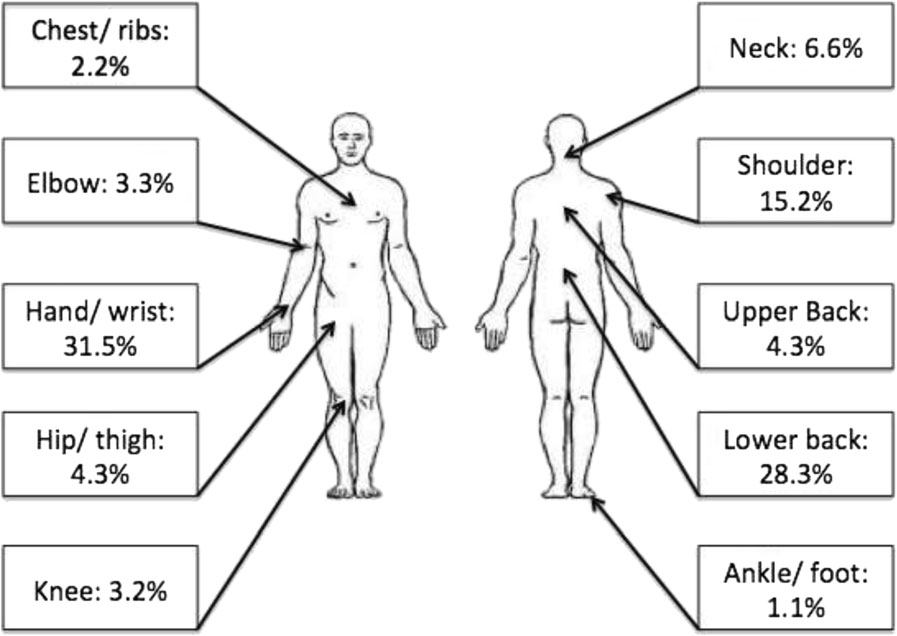
Bodyparts injured

Forty-two chiropractors (69%) reported experiencing a total of 92 injuries at 10 anatomical sites arising while working as a chiropractor/or prior injury aggravated by the profession (42/61). A higher prevalence of WRMSI was found in females. Injuries to the upper extremity were most commonly reported (Fig. 1 and Additional file 2), including hand/ wrist (31.5%) (*p* = 0.002) and shoulder (15.2%). Lower back injuries were reported by 28.3% of the injured chiropractors. The majority of the injuries involved soft tissue (Table 1), including ligament sprains (27.5%) (*p* = 0.150), muscle strain (26.6%) (*p* = 0.043) and tendonitis (14.7%) (*p* = 0.305). It was noted that 4.6% of injuries affected intervertebral discs and 2.8% of the injuries caused neuropathy. Most injuries reported were from cumulative trauma (43.8%) or an initial episode at work/ outside of work with subsequent flare-ups (32.58%). Most of the injuries occurred while either performing (38.2%) or positioning (10.11%) a patient for manipulation (*p* = 0.002) and maintaining a prolonged position (14.6%). The most common areas chiropractors were manipulating when injury occurred were the lumbar spine (57.7%), sacro-iliac joint (23%) and the thoracic spine (11.5%). Most commonly injuries occurred while the patient was being manipulated in the side-lying position (61.5%) (*p* = 0.021).

**Table 1:** Injury types

Injury types	Percentage of chiropractors with injuries (%) (N)
Ligament sprain	27.5% (*n* = 16.7)
Ligament tear	4.6% (*n* = 2.8)
Muscle strain	26.6% (*n* = 16.2)
Synovitis	3.7% (*n* = 2.3)
Tendonitis	14.7% (*n* = 8.9)
Dislocation	0% (*n* = 0)
Fracture	1.8% (*n* = 1.1)
Neuropathy	2.8% (*n* = 1.7)
Vertebral disc	4.6% (*n* = 2.8)
Other	13.8% (*n* = 8.4)

**Fig. 2:**
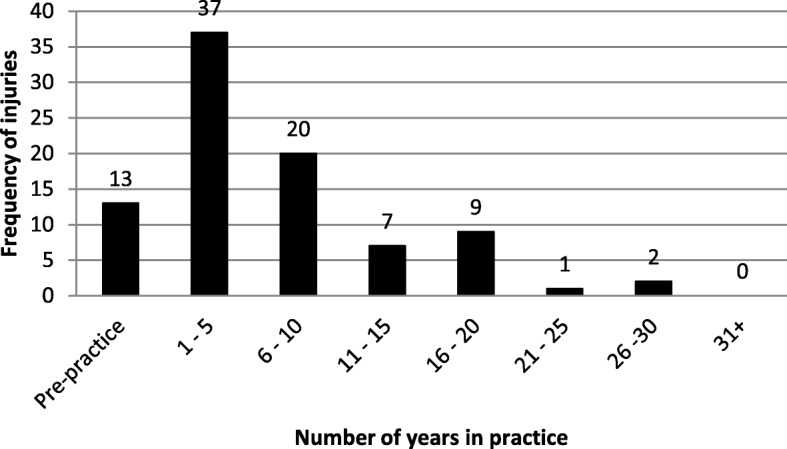
Year in practice when injury occurred

The majority of injuries (41.6%) occurred within the first to fifth year of practice (*p* = 0.032) (Fig. 2). Of note, 14.6% of injuries occurred while the chiropractors were still in training. In general (78.7%) respondents did not need to take any time from practice as a result of the injury. However, 9% of the injuries required the chiropractor to take one or more week’s leave from their practice, while 5.6% are still suffering with the injury.

**Fig. 3:**
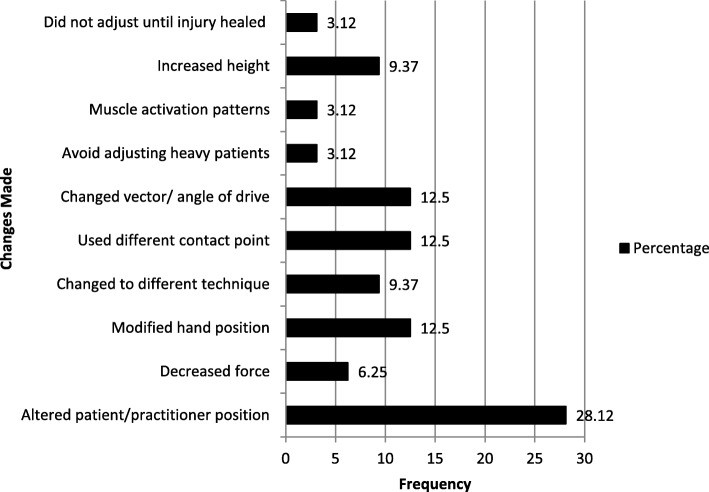
Changes made following injury

Thirteen chiropractors (32.5%) indicated that they implemented changes after their most severe work related musculoskeletal injury (Fig. 3). The most common changes were altering patient/ chiropractor position (28.1%), modified hand position (12.5%), use a different contact point (12.5%), switched to an alternative manipulation technique (12.5%).

With regards to income protection, 70% of respondents indicated they had income protection, however only a small percentage of respondents claimed from income protection.

## Discussion

WRMSI are a significant issue in the health care sector. Literature both international and local show many comparisons to this current study [45, 60–62]. Research by physiotherapist, osteopaths and chiropractors show common WRMSI to the following areas ie: the low back, neck, shoulder hand and wrist [45, 60–62]. Another common finding most WRMSI are under-reported due to dedication to patient care and financial constraints [45, 60–62].

The response rates are calculated by dividing the number of usable responses returned by the total number eligible in the sample chosen [63]. The response rate of 64% was much higher than 30% reported by Rupert and Ebete [39] and the 42.2% reported by Holm and Rose [45]. The majority of respondents (24%) were between the ages of 31 and 40 years; followed by 29.5% being between the ages of 25-30 years of age. Ages ranged from 25 to 69 years. The average age was 35.5 years. This is in keeping with previous studies conducted in South Africa where the majority of chiropractors showed a tendency towards ages 25 – 38 years [34, 46, 64–67]. Fyfe [34] found the mean age of chiropractic students to be 22.7 years (SD 3.5 years), ages ranged from 18 to 37 years which could explain the majority of respondents being between the ages of 31–40 years of age.

This could be in light of the fact the chiropractic is a relative new profession in South Africa, with the first intake of students in 1989 which graduated in 1994. Preceding this, a chiropractic qualification could only be acquired abroad. In comparison to American based studies which showed a slightly higher mean age of chiropractors ranging from 41 to 46 years [44, 45] which could be attributable to the fact that the first chiropractic graduates were produced much earlier.

The majority of participants were female (55.7%) therefore this sample was considerably different from previous studies carried out on chiropractors in South Africa in which the samples were predominantly male [46, 64–67]. It was not possible to determine the male to female ratio of chiropractors in South Africa as their gender was not specified on the Allied Health Professions Councils register. The results of this study differs from international studies which showed a male predominance in the chiropractic profession [39, 44, 45].

Historically the chiropractic profession was male dominated. However, it would seem that the ratio of men to woman is gradually starting to even out due to an increasing number of women qualifying as chiropractors. According to the National Board of Chiropractic Examiners 72.9% of chiropractic practitioners were male and 27.1% were female in a practice analysis survey completed in 2014. This further illustrates an increase in females if compared to a similar practice analysis done on 1991 where it was shown that 86.7% were male [68].

The sample in this study had a high prevalence of White participants (77%) with 21.3% being Indian and only 1.6% being African. These results were not surprising as the chiropractic profession is not well represented by the African population in South Africa or abroad [68, 69]. NBCE [68] indicated Africans only represented a small percentage (1.2%) of the chiropractors in the United States of America. In South Africa previous studies concur with these findings [46, 66, 67].

The average time that the respondents have been in practice was 9.44 years. These figures correlate with previous South African studies which portrayed the majority of South African chiropractors have spent less than 10 years in practice [46, 47, 64–66]. These results differ from international studies which indicated the majority of chiropractors have been in practice for 16.4 years [45].

The majority of respondents spent between 31 and 40 h in clinical practice per week which coincides with previous studies done on South African chiropractors as well as international studies [47, 66–68].

The average number of patient seen per day varied between 6 and 10; which coincides with the average of nine patient per day cited by Pereira [47]; however these figures are slightly less than the 11–20 cited by Mathews [46]. If the figures of this study were to be extrapolated to patients seen per week it would equate to roughly 30–50 patient per week (on an average 5 day week).

Large inconsistencies exist when these figures are compared to international studies. Holm and Rose [45] reported a mean practice volume of 114 patient per week while NBCE [68] stated the majority of chiropractors treat between 50 and 99 patients per week.

The time spent with patients was roughly estimated with the majority of respondents spending 36–40 h per week in clinical practice it would roughly equate to 8 h per day, 5 days per week. If this s to be divided by 10 patients seen per day would equal a crude estimate of 45–60 min spent per patient. In contrast to American chiropractors whom also spend 40 h per week in clinical practice seeing 20 patients per day would equate to 24mins spent per patient if 99 patients were seen per week. According to the study done by Holm and Rose [45], 114 patients were seen per week which would approximately equate to 23 patients seen per day with 21 min spent per patient.

South African chiropractors might have a lower practice volume in comparison to chiropractors overseas; which should protect them against WRMSKI as high practice volume has been identified as a risk factor for the development of WRMSKI [69]. However the results found in this study stands in contrast to this as 68.9% of chiropractors indicated they have suffered from a WRMSKI as opposed to the 40.1% reported by Holm and Rose [45].

A study conducted by Cromie, Robertson and Best [69] correlated the prevalence of thumb pain to the hours worked per week in physiotherapists and concluded that these symptoms increased in a linear relationship to the hours worked per week.

The high prevalence might be attributed to the fact that South African chiropractors spend more time with their patients in the clinical setting.

Questions pertaining to the manipulative technique utilised by the practitioner on a daily basis the majority of practitioners indicated Diversified technique (91.8%) was used on every patient or regularly as treatment technique. This is in line with previous South African studies which showed Diversified as the most used [46, 65–67] as well as international studies done [45].

The Diversified technique was most commonly used which was expected seeing the Diversified technique is taught in the curriculum at both Chiropractic schools in South Africa (Durban University of Technology 2017; University of Johannesburg 2017).

The majority of respondents (75.4%) indicated they use dry needling (75.4%) either on every patient or regularly as adjunct treatment to manipulation. This is consistent with findings reported by De Gouveia [65] and Keyter [66] which indicated that dry needling was one of the most utilised modalities in practice. Whereas 60.7% indicted they used massage on very patient or regularly which is slightly less than the 81.5% cited by De Gouveia [65], but higher than the 43.6% reported by Gordon [67].

The practice lifetime prevalence of WRMSKI in chiropractors in the eThekwini municipality was 69.85%. When compared to similar studies relatively large differences are noted between the studies. Holm and Rose [45] reported a prevalence of 40.1% (*n* = 397) whereas Homack [44] reported 84% (*n* = 72) of chiropractors have sustained a WRMSKI.

This study found a slighly higher prevalance of WRMSKI amongst female respondents, however this could be due to the higher percentage of female participants in this study. Homack and Hedge [70] reported a male dominace (57.9%) with respect to injuries reported. This is supported by Holm and Rose [45] who found 95.2% of practitioners who reported three injuries were male.

The upper extremity was most vulnerable to WRMSKI especially the hand/ wrist, followed by the low back. Hand/ wrist injuries could be ascribed to the technique used when manipulating patients. Placing the wrist in either flexion, extension, radial or ulnar deviation was found to be a risk factor for developing WRMSKI, this possibly coupled with incorrect placement or inflexibility of the wrist during manipulative procedures, could further cause biomechanical strain on the joints and soft tissue of the hand and wrist. Manipulation requires the wrist to be placed in a combination of the above mentioned positions which predisposes the hand and wrist to injury [17, 45].

Most injuries involved the soft tissue which correlate with previous international studies [45, 70]. Scar tissue is less elastic in nature with more collagenic properties, by altering the properties of the tissue the range of future use is invariably limited and increases the susceptibility to future injury [23] which explains the high prevalence of injury caused by cumulative trauma.

The most injuries occurred with manipulation of the lumbosacral spine with patients in the side lying posture. These results can be attribute to the fact that the majority of lumbosacral manipulations with the patient in a side lying position requires the chiropractor to assume a forward flexion position with a certain degree of trunk rotation. There is strong evidence in the literature that suggests low back injuries is the consequence of awkward work postures includin non-neutral postures relating to forward flexion and trunk rotation [4, 59]. Maintaining static posture for prolonged periods of time causes static loading of the muscles which has been causally linked to the development of low back pain [59].

Difficult to report on the technique used as the majority of chiropractors use more than one technique however diversified was most commonly cited/ reported as technique used when injured.

The majority of injuries occurred within the first 5 years of practice. This is supported by previous South African studies. Mathews [46] investigated the prevalence of occupational overuse of the hands and wrists and reported a mean onset of 3.41 years. Another study conducted by Pereira [47] found chiropractors experience low back pain for the first time within the first 5 years of practice. Holm and Rose [45] also reported similar findings; literature in the physiotherapy realm supports comparable findings [51, 69, 71–73]. Greene, Goggins and Hess [52] stated that previous musculoskeletal injuries is a strong predictor for future injury which explains why the majority of injuries occurred within the first 5 years of practice as results in this study found 14.6% of the injuries occurred pre-practice. These results are in line with the study done by Ndetan et al. [48] who investigated injuries in chiropractic students and found that 30.95% (13/42) of the students sustained an injury pre-practice (i.e. while being a student), they ascribed the high prevalence of injuries in students due to lack of experience while receiving and applying manipulations. This literature can be used to infer that the majority of newly graduated chiropractors are not using ideal biomechanics when manipulating patients.

Although there was a large number of WRMSKI reported, only a few of the respondents indicated they had taken time off practice following the injury. These results correspond to results found by Holm and Rose [45] which indicated 69.8% of chiropractors did not require any time off practice as well as findings reported by Darragh, Huddleston and King [74] which showed almost all occupational and physical therapists who reported work-related injuries continued working.

Chiropractor are less likely to seek care, take time off or file a worker’s compensations claim because of the ability to self-treat, recognise early symptoms of injury [75]. Chiropractors may self-treat symptoms, use colleagues or self-prescribe treatment programmes [73, 75]. Another plausible reason for the lack of time taken off practice could be that he majority of practitioners being sole proprietors. The questionnaire did not ask the chiropractor to divulge whether they are sole proprietors or hold an associated/partnership position. However gleaned from a practice analysis done in America in 2015 it was reported that 74.4% of chiropractors were sole proprietors [68], which could explain the lack of time taken off from practice due to the injury.

The greater majority of respondents indicated that they made no change to their practice following the injuries, only 33.33% indicated they made changes following the injury which agrees with results found by Holm and Rose [45].

The most common changes included modification of patient or practitioner posture/position and modification of hand position which is similar to changes described by Holm and Rose [45].

Although the majority of respondents who suffered from WRMSKI had income protection (70%) only a small number of them claimed due to the injury. Only six cases of claims were reported of which all six claims were paid out successfully. According to PPS (2013) [76] 20% of all claims were due to musculoskeletal and connective tissue disorders.

### Limitations

South Africa is considered as a developing or third world country, the profession of chiropractic is relatively new to the majority population that belong to a below average socio economic background. Furthermore Chiropractic treatment is not offered in provincial state hospitals thus only being available to private health patients. Chiropractic treatment for this reason is considered a luxury treatment within these poor socioeconomic settings. Therefore the majority South African population requires education on the profession of Chiropractic. Hence, the in-take of students at chiropractic institutions are few, resulting in the production of a small number of chiropractors. This thus contributes to the small population of chiropractors available as a sample population for research conducted in South Africa. Due to this study, being restricted to only chiropractors practicing within the eThekwini municipality, results in a further reduction in the available sample population. Although a satisfactory response rate of 64% was achieved, future studies should aim to investigate a broader scope of practitioners in Kwa-Zulu Natal and nationally. This would ensure that the study could represent the entire chiropractic population adequately.

Chiropractors who have left the profession due to permanent disability were not included in this study neither were chiropractors that were on extended leave (i.e. maternity leave) at the time of the questionnaire.

The ability to accurately recall injuries that may have occurred a long time ago (mean number of years spent in practice in this study was 9.43 years) is another limitation to the internal validity of the study.

## Recommendations

In a study of this nature, the researcher relies on the respondents to have answered the questionnaire openly and honestly, therefore allowing the research to be the best approximation of work-related musculoskeletal injuries incurred by respondents. The outcomes of this study only include information from chiropractors that accepted the invitation to participate in this study, thus the results may only be generalised to similar population groups.

Future studies should consider adding questions pertaining to:

- The type of practice the participants worked in (e.g. solo, associate/ partner) which could have influenced the ability to take time off work. Sole proprietor might be less inclined to take time off.
- The use of a height adjustable treatment table was suggested/ implemented.

## Conclusion

This study determined that injuries to the upper limb and lower back were more prevalent than injuries to other anatomical regions. The hand/wrist was the most common anatomical site of injury in chiropractors, followed by the lower back. The majority of injuries affected the soft tissue, including muscle strains and ligament sprains.

Factors that increased the likelihood of a chiropractor sustaining a work-related musculoskeletal injury included the use of the diversified technique, particularly with the patient in the side lying position to manipulate the lumbosacral area.

Most injuries occurred within the first 5 years of practice and were related to cumulative trauma. However, only a third of chiropractors indicated they had made changes to their practice as a result of the injury.

Based upon the conclusion of this study, there is a need for preventative programmes and safe practice guidelines for chiropractors - especially intervention services designed to reduce the rate of work-related musculoskeletal pain among newly graduated practitioners.

## Additional files

Additional file 1: Appendix A: Letter of information. Appendix B: Informed consent. Appendix C: Research questionnaire. (DOCX 356 kb)

Additional file 2: Data: Body map. Percentage of body part injured. (PDF 216 kb)
