# Supplementary material for: The epidemiology of work-related musculoskeletal injuries among chiropractors in the eThekwini municipality
Source: Chiropr Man Therap. 2019 Mar 19;27:18. doi: 10.1186/s12998-019-0238-y (PMC6423772; doi:10.1186/s12998-019-0238-y)
Supplement: Supplementary file 1 — Appendix A: Letter of information. Appendix B: Informed consent. Appendix C: Research questionnaire. (DOCX 356 kb) [file 12998_2019_238_MOESM1_ESM.docx]

Appendix A

**LETTER OF INFORMATION**

Dear Participant, I would like to welcome you into the research study.

**Research Title:**

The epidemiology of work-related musculoskeletal injuries among chiropractors in the eThekwini municipality

**Supervisor:** Dr. K. Padayachy, M.Tech Chiropractic , PhD ( Anatomy)

**Research student:** Almay Lamprecht, B.Tech Chiropractic

**Brief Introduction and Purpose of the Study:**

There is a higher prevalence of work-related musculoskeletal injuries in health care workers when compared to other sectors due to their labour-intensive and physically demanding work-activities. Patient handling (including patient transfers, repositioning and lifting) and manual therapy (soft tissue work, mobilisation of joints and orthopaedic techniques) are activities most commonly cited in association with work-related musculoskeletal injuries among health care professionals.

There is paucity in the literature on the epidemiology of work-related musculoskeletal injuries in chiropractors focusing on the entire chiropractic practitioner.

Participation is voluntary and refusal to participate in this study will not result in adverse consequences of any kind. The research manuscript will be made available at the Durban University of Technology Library, in the form of a mini-dissertation and results as seen in the research abstract will be sent to you should you request it.

**Outline of the Procedures:** You will receive a letter of information and if you agree to participate in the study, you will be required to complete and sign a letter of informed consent. You will then be required to complete the research questionnaire, which may take on average about 10-15 minutes.

Please note: you are free to withdraw from this research study at any time without giving a reason.

**Benefits:** By determining the epidemiology of work-related musculoskeletal injuries in chiropractors in eThekwini municipality - the prevalence, associated risk factors and causal features can be recognised and employed to create strategies to prevent/lessen work-related musculoskeletal injuries in the chiropractic profession. This will impact the practitioner beneficially while the patient has the advantage of receiving optimal care from their chiropractor.

**Remuneration:** There will be no monetary remuneration or costs for undertaking this study by any of the participants in this study.

**Costs of the Study:**There is no cost involved for your participation in this study. The questionnaire and related information will be sent to you via email, once done please email the completed questionnaire to [WRMSIquestionnaire@gmail.com](mailto:wrmskiquestionnaire@gmail.com). If you elected to receive a hard copy of the questionnaire the questionnaire is hand delivered and taken on completion.

**Confidentiality:** Your participation is completely voluntary and all responses will be treated confidentially and results will be used for research purposes only. Completed questionnaires and consent forms will be received by the researcher who will separate the informed consent from the completed questionnaire after which participants will be coded to ensure confidentiality.

**Persons to Contact in the Event of Any Problems or Queries:** Please contact the researcher ([WRMSIquestionnaire@gmail.com](mailto:wrmskiquestionnaire@gmail.com)), my supervisor; Dr. K. Padayachy ([keserip@hotmail.com](mailto:keserip@hotmail.com)) or the Institutional Research Ethics Administrator on 031 373 2900. Complaints can be reported to the Director: Research and Postgraduate Support, Prof S Moyo on 031 373 2577 or [moyos@dut.ac.za](mailto:moyos@dut.ac.za)

Thank you for your participation and cooperation.

Your time and assistance with this project is invaluable and is greatly appreciated.

**Appendix B**

**INFORMED CONSENT**

Statement of Agreement to Participate in the Research Study:

I hereby confirm that I have been informed by the researcher, Almay Lamprecht about the nature, conduct, benefits and risks of this study (IREC Reference Number: REC 61/16)

I have also received, read and understood the above written information (Participant Letter of Information) regarding the study.

I am aware that the results of the study, including personal details regarding my sex, age, date of birth, initials and diagnosis will be anonymously processed into a study report.

In view of the requirements of research, I agree that the data collected during this study can be processed in a computerised system by the researcher.

I may, at any stage, without prejudice, withdraw my consent and participation in the study.

I have had sufficient opportunity to ask questions and (of my own free will) declare myself prepared to participate in the study.

I understand that significant new findings developed during the course of this research which may relate to my participation will be made available to me.

Full Name of Participant

Date and Time

Tick box for informed consent ☐

I, Almay Lamprecht herewith confirm that the above participant has been fully informed about the nature, conduct and risks of the above study.

**Full Name of Researcher Date Signature**

# APPENDIX C

## RESEARCH QUESTIONNAIRE

1. **Demographic detail (Tick appropriate box)**
   1. Gender
   2. Age in years 00
   3. Race (for statistical purposes only)

(If other, please specify)

- 1. Height in meters 0.00
  2. Weight in kilogrammes 000

1. **Practice demographics**
   1. Time in practice (please specify number of years) 00
   2. The average number of patients seen per working day? 00
   3. Average number of hours spent in clinical practice per week (hands on work) 00,0

| Activator |  |  |  |  |  |
| --- | --- | --- | --- | --- | --- |
| Diversified |  |  |  |  |  |
| Gonstead technique |  |  |  |  |  |
| NIP |  |  |  |  |  |
| SOT |  |  |  |  |  |
| Thompson technique |  |  |  |  |  |

- 1. Chiropractic technique/s used on a daily basis **(Tick appropriate box)**?

Other (please specify)

- 1. Non-manipulative techniques used on a daily basis (tick appropriate box indicating frequency of each option used):

| Dry needling/ acupressure |  |  |  |  |  |
| --- | --- | --- | --- | --- | --- |
| Electro-modalities |  |  |  |  |  |
| Ice/ heat packs |  |  |  |  |  |
| Massage therapy |  |  |  |  |  |
| None |  |  |  |  |  |
| Strapping |  |  |  |  |  |
| Stretching |  |  |  |  |  |

Other (please specify)

- 1. Have you experienced a work-related musculoskeletal injury arising out of employment as a chiropractor/or prior injury aggravated by your profession?

1. **Work related musculoskeletal injuries: SINGLE MOST SEVERE work related musculoskeletal injury**
   1. What body part was affected? **(Tick appropriate box)**

- 1. What type of injury was it (check all that apply)

Ligament sprain/ strain ☐ Dislocation ☐

Ligament / tendon tear ☐ Fracture ☐

Muscle strain ☐ Neuropathy ☐

Synovitis ☐ Vertebral disc injury ☐

Tendinitis ☐ Other ☐

If other, please specify)

- 1. Was this injury a result of**:**

(Please specify how injury occurred)

- 1. Activity you were performing that caused your injury/ aggravated existing injury?

If other, please specify

- - 1. If injury occurred during manipulation:
       1. What area were you adjusting?
       2. What technique did you use?
       3. Patient position in which you adjusted?

- 1. Year of practice in which injury occurred ( or 1^st^ occurred if repetitious / chronic injury):

- 1. How much time were you away from work due to this injury to date?

(If still suffering, please specify)

- 1. Did you change your adjusting technique(s) as a result of the injury?

If YES, what did you change?

- 1. What other changes, if any, did you make to your practice as a result of this injury (be specific e.g. environmental/ treatment technique etc.)

- 1. Do you have Income Protection?

- 1. Did you claim for this injury

- 1. Did your claim pay out?

1. **Work related musculoskeletal injuries: SECOND MOST SEVERE work related musculoskeletal injury**
   1. What body part was affected? **(Tick appropriate box)**

- 1. What type of injury was it (check all that apply)

Ligament sprain/ strain ☐ Dislocation ☐

Ligament / tendon tear ☐ Fracture ☐

Muscle strain ☐ Neuropathy ☐

Synovitis ☐ Vertebral disc injury ☐

Tendinitis ☐ Other ☐

If other, please specify)

- 1. Was this injury a result of:

(Please specify how injury occurred)

- 1. Activity you were performing that caused your injury/ aggravated existing injury?

If other, please specify

- - 1. If injury occurred during manipulation:
       1. What area were you adjusting?
       2. What technique did you use?
       3. Patient position in which you adjusted?

- 1. Year of practice in which injury occurred ( or 1^st^ occurred if repetitious / chronic injury):

- 1. How much time were you away from work due to this injury to date?

(If still suffering, please specify)

- 1. Did you change your adjusting technique(s) as a result of the injury?

If YES, what did you change?

- 1. What other changes, if any, did you make to your practice as a result of this injury (be specific e.g. environmental/ treatment technique etc.)

- 1. Do you have Income Protection?

- 1. Did you claim for this injury

- 1. Did your claim pay out?

1. **Work related musculoskeletal injuries: THIRD MOST SEVERE work related musculoskeletal injury**
   1. What body part was affected? **(Tick appropriate box)**

- 1. What type of injury was it (check all that apply)

Ligament sprain/ strain ☐ Dislocation ☐

Ligament / tendon tear ☐ Fracture ☐

Muscle strain ☐ Neuropathy ☐

Synovitis ☐ Vertebral disc injury ☐

Tendinitis ☐ Other ☐

If other, please specify)

- 1. Was this injury a result of:

(Please specify how injury occurred)

- 1. Activity you were performing that caused your injury/ aggravated existing injury?

If other, please specify

- - 1. If injury occurred during manipulation:
       1. What area were you adjusting?
       2. What technique did you use?
       3. Patient position in which you adjusted?

- 1. Year of practice in which injury occurred ( or 1^st^ occurred if repetitious / chronic injury):

- 1. How much time were you away from work due to this injury to date?

(If still suffering, please specify)

- 1. Did you change your adjusting technique(s) as a result of the injury?

If YES, what did you change?

- 1. What other changes, if any, did you make to your practice as a result of this injury (be specific e.g. environmental/ treatment technique etc.)

- 1. Do you have Income Protection?

- 1. Did you claim for this injury

- 1. Did your claim pay out?

**Thank you for your time**

**Kind regards**
