# Supplementary figures and images for: The epidemiology of work-related musculoskeletal injuries among chiropractors in the eThekwini municipality
Source: Chiropr Man Therap. 2019 Mar 19;27:18. doi: 10.1186/s12998-019-0238-y (PMC6423772; doi:10.1186/s12998-019-0238-y)

Additional file: Body Map

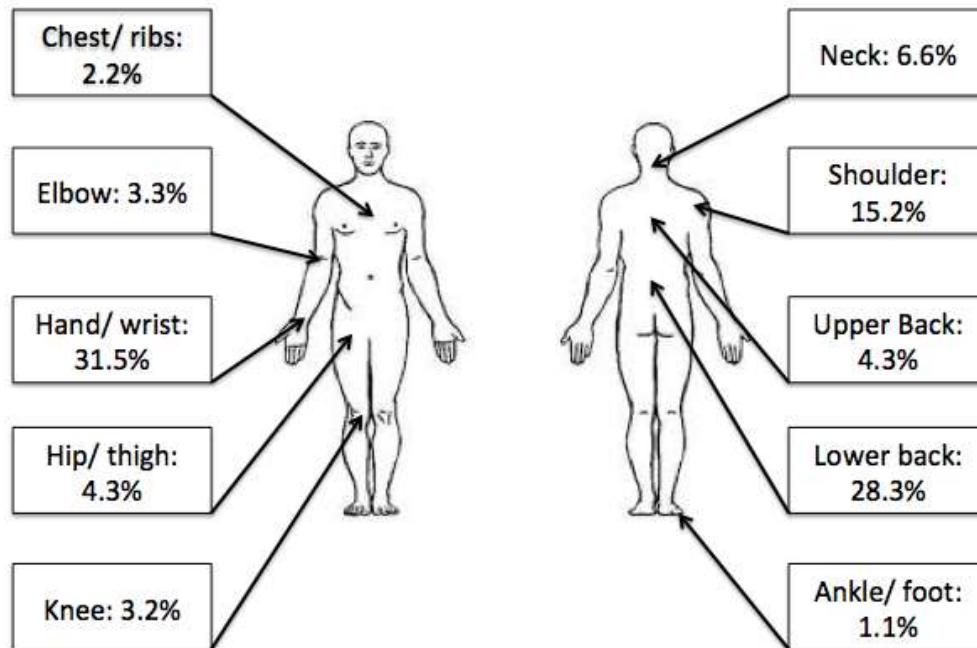

Supplement: Supplementary file 2 — Data: Body map. Percentage of body part injured. (PDF 216 kb) [file 12998_2019_238_MOESM2_ESM.pdf]
